# Mitochondrial Dysfunctions and Altered Metals Homeostasis: New Weapons to Counteract HCV-Related Oxidative Stress

**DOI:** 10.1155/2013/971024

**Published:** 2013-11-24

**Authors:** Mario Arciello, Manuele Gori, Clara Balsano

**Affiliations:** ^1^Department of Internal Medicine and Medical Specialties, “Sapienza” University of Rome, Via del Policlinico 155, 00161 Rome, Italy; ^2^Francesco Balsano Foundation, Via G.B. Martini 6, 00198 Rome, Italy; ^3^Institute of Molecular Biology and Pathology (IBPM); CNR, Piazzale Aldo Moro 7, 00185 Rome, Italy

## Abstract

The hepatitis C virus (HCV) infection produces several pathological effects in host organism through a wide number of molecular/metabolic pathways. Today it is worldwide accepted that oxidative stress actively participates in HCV pathology, even if the antioxidant therapies adopted until now were scarcely effective. HCV causes oxidative stress by a variety of processes, such as activation of prooxidant enzymes, weakening of antioxidant defenses, organelle damage, and metals unbalance. A focal point, in HCV-related oxidative stress onset, is the mitochondrial failure. These organelles, known to be the “power plants” of cells, have a central role in energy production, metabolism, and metals homeostasis, mainly copper and iron. Furthermore, mitochondria are direct viral targets, because many HCV proteins associate with them. They are the main intracellular free radicals producers and targets. Mitochondrial dysfunctions play a key role in the metal imbalance. This event, today overlooked, is involved in oxidative stress exacerbation and may play a role in HCV life cycle. In this review, we summarize the role of mitochondria and metals in HCV-related oxidative stress, highlighting the need to consider their deregulation in the HCV-related liver damage and in the antiviral management of patients.

## 1. Introduction

Hepatitis C virus (HCV) is a human pathogen affecting about 4 million new subjects every year [1]. Approximately 3% of the world's population is estimated to be chronically infected by HCV [2]. Differently from the other hepatitis viruses (A, B, and E), more than 80% of HCV patients become chronic [3].

HCV is a member of the genus *Hepacivirus* of Flaviviridae family. It is a single-stranded RNA virus with positive polarity. The genome of HCV encodes a polyprotein of about 3000 amino acids that is expressed from a single long open reading frame (ORF). This polyprotein is cleaved into ten different products: the core protein (Core) and the envelope glycoproteins 1 and 2 (E1 and E2, resp.), which are constituents of the HCV particles, p7 and nonstructural protein 2 (NS2), primarily involved in HCV assembly, NS3, NS4A, NS4B, NS5A, and NS5B nonstructural proteins with important roles in the polyprotein processing and HCV replication [4]. HCV infection frequently leads to severe liver diseases, including liver cirrhosis and HCC [5]. Chronic HCV infected patients are commonly characterized by metabolic derangements, such as steatosis, insulin resistance (IR), and altered homeostasis of trace metals [6–8]. Many works suggest that oxidative stress (OS) plays a pivotal role in the occurrence of all these pathological features. OS is the condition occurring when the cellular or systemic redox balance is altered, as a consequence of unusual exposure to prooxidant molecules, like reactive oxygen species (ROS) or reactive nitrogen species (RNS) [9], which in turn can be either associated with an inadequate antioxidant response or not. The overproduction of ROS and RNS can be caused either by endogenous or exogenous sources [9]. OS produces oxidative damage to proteins, lipids, and nucleic acids, thus altering their physiological functions.

Mitochondria are the main source of ROS production through the electron transport chain (ETC) complexes and the mitochondrial dehydrogenases [10] and, at the same time, they are the main targets of reactive molecules. Mitochondria are well-known targets of HCV protein actions; however, also extramitochondrial sources of ROS are involved in HCV-related OS onset: ER, peroxisomes and other cell compartments [11, 12], xanthine oxidase or NADPH oxidases [13], cytochromes P450, and resident immune cell populations in the liver (e.g., Kupffer cells). To avoid the deleterious effects of ROS, biological systems have developed several mechanisms of detoxification that use a wide number of small molecules, peptides, and enzymes, like glutathione (GSH) or superoxide dismutases (SODs), respectively.

However, it should not be forgotten that ROS are also potent second messengers in a plethora of cellular functions; they are involved in modulating key physiopathological processes [14, 15], such as those mediated by the signal transducer and activator of transcription (STAT) and nuclear factor kappa-light-chain enhancer of activated B cells (NF*κ*B) [16].

This review will summarize some relevant mechanisms through which HCV may promote OS onset, focusing the attention on virus-related mitochondrial OS, trace elements derangement, and novel therapeutic opportunities to counteract these viral actions.

## 2. Oxidative Stress during HCV Infection

The OS occurrence, as a consequence of HCV infection, is well established [17]. Several reports put in evidence a deep redox imbalance in infected patients, focusing on its potential correlation with the course of the liver disease.

Increased markers of oxidative-damaged DNA (8-hydroxyguanosine, 8-OHdG) and lipid peroxidation products (8-isoprostane) were detected in serum, peripheral blood mononuclear cells (PBMC), and liver specimens of infected patients [5, 18, 19].

Accordingly, we recently demonstrated that HCV patients are characterized by increased plasma levels of 7-ketocholesterol (7K) and 7-*β*-hydroxycholesterol (7*β*OH) [20], two of the well-known peroxidation products of cholesterol, called oxysterols. Oxysterols are extremely interesting, not only because they can modulate the mitogen-activated protein kinases (MAPKs), thus affecting cell growth and promoting cell transformation [21], but also because they were detected in oxidized-low density lipoprotein (oxLDL) [22]. Oxysterols are the specific ligands of liver X receptors (LXRs), nuclear receptors deeply involved in several pathophysiological processes, for example, lipid metabolism and inflammation [23]; through oxysterols HCV might profoundly modulate host metabolism. Accordingly, steatosis is a common feature in HCV-infected hepatocytes [24] and it is characterized by high oxidation rates with a consequent increase of electron delivery to the ETC that might cause ROS overproduction. Infected patients show a correlation between OS markers and inflammation, grade of fibrosis, and hepatic iron storage [25, 26]. However, the increased levels of oxysterols give rise to the question whether the OS is directly due to HCV-host cell interactions or if host immune response and iron overload are main reasons for OS onset [27–29]. Surely, a synergistic mechanism exists.

Moreover, HCV patients are deeply characterized by a reduction of their antioxidant defense. Glutathione (GSH), a key player of the first line of antioxidant defense, produced by all cells and especially concentrated in liver [30], is commonly decreased in chronic HCV subjects [31–33]. Accordingly, the ratio between oxidized (GSSG) and reduced (GSH) forms, a well-accepted parameter representative of the oxidative status, is increased [32]. As well as GSH, glutathione reductase, glutathione peroxidase, and Cu/Zn containing SOD are often found decreased in PBMC of infected patients too [34].

All reported data demonstrate that OS occurrence is due to HCV, but some discrepancies exist about the entity of the induction of liver damage linked to this pathological mechanism. To note, OS markers are found in HCV patients with mild, moderate, or no liver disease [35]. However, proteomic analysis revealed an upregulation of antioxidant enzymes at early (F1 to F3) but not at late stages of fibrosis [36].

On the contrary, some works described an enhanced expression of thioredoxin (Trx) [37] or heme oxygenase (HO-1) [38]. The latter enzyme, in particular, is a known target gene of the nuclear factor erythroid2-like 2 (Nrf2) protein. This data seems in contrast with what we have previously described, but, in our opinion, the activation of Nrf2 could be functional to HCV virus. In fact, Nrf2 is a transcription factor which recognizes a common conservative sequence, called antioxidant response element (ARE), in the promoter regions of many antioxidant enzymes [39, 40], and is crucial to preserve the mitochondrial activities and to enhance cell survival of infected cells [41]. Thus is essential for viral life cycle.

## 3. Mitochondria: In the Center of the Viewfinder of HCV

As mentioned above, the main parts of ROS produced in the cells are generated by mitochondria. These organelles are the “cellular power plants,” because they are mainly responsible for cell supply of adenosine triphosphate (ATP). By sensing the energy status they may decide the cell fate [42]. The relevance of mitochondria to metabolism, energy production, and cell fate was brought to light by several reports in a plethora of human diseases, from neurodegeneration to metabolic disorders [43].

Several lines of evidence describe, without any doubt, mitochondria as a main target of the HCV virus. It is well known that HCV produces ultrastructural alteration of these organelles and causes oxidative damage and a reduction in mitochondrial DNA copy number, in both hepatocytes and lymphocytes of infected patients [44, 45].

Mitochondria possess a complex architecture; they comprise an outer membrane (OMM) that encloses the entire organelle and has a structure similar to that of plasma membrane. The OMM contains proteins and complexes that allow diffusion of small proteins or factors characterized by a specific signaling sequence at their N-terminus. The OMM can associate with the ER through a structure called mitochondria-associated ER membrane (MAM) [46]. MAMs are zones of junctions where the inner mitochondrial membrane (IMM) meets the OMM and allows exchanges of Ca^2+^ and lipid between ER and mitochondria [46]. Thus, MAMs are key elements in the maintenance of mitochondrial homeostasis of lipids and Ca^2+^ [46] and are crucial to modulating the opening of the mitochondrial permeability transition pore (mPTP). The latter is a critical event in the decision of cell fate [47]. Furthermore, MAMs have a key role in the cellular pathways leading to very-low-density lipoprotein (VLDL) assembly and secretion [48]. These cellular activities are also essential for the formation and maturation of HCV particles [49].

**Figure 1:**
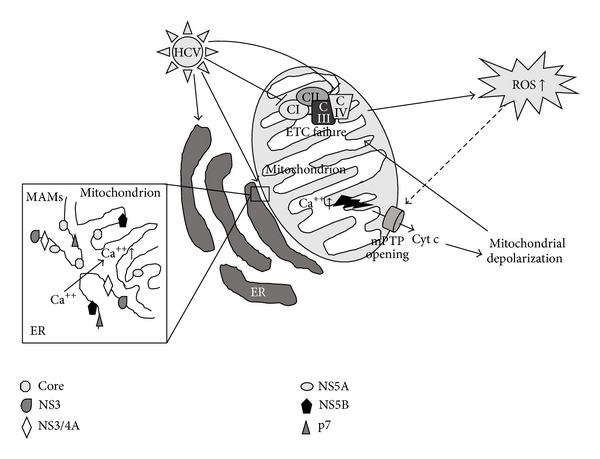
Molecular mechanisms through which HCV induces mitochondrial damage and the consequent increased ROS production. Several HCV proteins associate with both endoplasmic reticulum (ER) and mitochondria. In particular, Core localizes also on MAMs and may play a role in the increase of mitochondrial Ca^++^ pool, which in turn is involved in mitochondrial permeability transition pore (mPTP) opening, release of cytochrome c (Cyt C), and consequent mitochondrial depolarization. Organelle depolarization may be responsible for electron transport chain (ETC) failure and reactive oxygen species (ROS) overproduction. Enhanced ROS, in turn, may cause a further mPTP opening (dashed line), worsening the mitochondrial depolarization, thus leading to the onset of a vicious deleterious circle that aggravates the mitochondrial damage. Furthermore, some reports indicate that Complexes I and IV (CI and CIV, resp.) of the ETC are main targets of HCV actions. The fall of their activities plays key role in ETC failure and increased ROS production.

Several HCV proteins have been shown to directly associate with mitochondria [50–52] (Figure 1). In particular, Core is able to associate with OMM through a specific sequence at its C-terminal region [53, 54]. It was detected in MAMs on the mitochondrial surface [53] and, by electronic microscopy; it was also localized in the IMM [52] (Figure 1). Biochemical studies revealed that the interaction of HCV with mitochondria, through the Core protein, plays a key role in organelles sensitization to Ca^2+^ influx, with the consequent opening of mPTP, release of cytochrome c [55], mitochondrial depolarization, and ETC failure. This cascade of events leads to an increased ROS generation (Figure 1).

Other viral proteins reinforce the Core action. Indeed, in experimental models, the proteins p7 and NS4A, the NS3/4A complex, and the proteins NS5A and NS5B were found to be localized to mitochondria and ER by subcellular-fractionation and confocal and electron microscopy [55–59] (Figure 1). Furthermore, both NS5A and NS5B were also localized in the IMM and in the mitochondrial matrix [55, 58] (Figure 1).

These data clearly demonstrate the intimate connection of HCV to mitochondria.

The mitochondrial sensitization to Ca^2+^ along with the mPTP opening, caused by viral proteins, lead to building up a vicious circle characterized by the inhibition of the ETC, with the consequent increase in ROS production. This, in turn, induces mPTP opening [60, 61] and further worsening of mitochondrial failure (Figure 1).

One of the main mitochondrial targets of HCV is Complex I of ETC (also known as NADH:ubiquinone oxidoreductase) [62]. Complex I activity, being the first step of ETC, is crucial for the aerobic respiration. Mitochondria isolated from transgenic mice expressing Core, E1, and E2 glycoproteins are characterized by an increased ROS production from Complex I substrates and reduced Complex I activity [17, 50] (Figure 1). The Complex IV (cytochrome c oxidase) is the other mitochondrial protein complex affected by HCV (Figure 1). On the contrary, Complexes II and III are not altered during HCV infection [63]. It is interesting to note that the inhibition of viral replication, *via* IFN treatment, can fully restore the activities of both Complexes I and IV in hepatic cells [63].

Increased oxidation of the GSH and thioredoxin pools further provides a demonstration of the HCV-related mitochondrial redox imbalance [64, 65]. Core has also been shown to induce the expression of mitochondrial but not cytoplasmic SOD [65], suggesting that HCV generates ROS at mitochondrial level and, at the same time, strengthens the cellular antioxidant system against OS. The virus, to avoid excessive cytotoxic effects produced by a massive increase of ROS, might activate this behavior.

## 4. HCV and Metals Homeostasis Derangement

HCV infection may prompt OS onset by deregulation of homeostasis of trace metals, like zinc (Zn), iron (Fe), and copper (Cu) [8]. Zn, Fe, and Cu are essential trace elements that play important roles in various biological processes.

HCV patients show low plasma concentrations of Zn, whereas Cu and Fe concentrations were high [8, 66]. Notably, higher amounts of both Fe and Cu can interfere with Zn homeostasis, worsening the Zn deficiency [67].

Zn is largely present in the cells [68] and has several relevant biological functions: it is involved in insulin management [69] and in the maintenance of immune system [70]. Zinc is a redox inert metal; thus, it does not directly participate in cellular reactions of reduction and oxidation. The antioxidant potential of Zn is exerted through different mechanisms: (a) it is able to bind the sulfhydryl groups of proteins avoiding their oxidation by free radicals; (b) it participates in the antioxidant response through the modulation of metallothioneins, GSH, and Nrf2; (c) it may antagonize redox-active transition metals, such as Fe and Cu [68]. Zn has also a structural relevance to HCV because some of its proteins, that is, NS3 and NS5A, are zinc metalloproteins [71]. At hepatic level, Zn is known to promote antioxidant and anti-inflammatory effects that result in reduced hepatocyte injury, in chronic HCV infected patients [72, 73]; moreover, Zn is able to inhibit NF*κ*B activation, thus counteracting the production of inflammatory cytokines [74]. HCV replication, instead, enhances the NF*κ*B pathway activation triggered by tumor necrosis factor-alpha (TNF*α*) [75]; thus the low Zn plasma levels may prompt the onset of an inflammatory environment known to play a key role in virus-related liver disease progression. Accordingly, some studies suggest that zinc administration, through a drug called polaprezinc, may improve the outcome in HCV and HCV-related cirrhotic patients [76, 77]. The idea that low levels of Zn may be functional to HCV-related liver damage seems further supported by some studies that have suggested its potential in inhibiting HCV replication [78]; however, the mechanism is still not clear.

As well as in other pathologies, such as cancer or diabetes [70, 79, 80], in the HCV infection a Zn deficiency occurs parallel to a Cu increase [8], particularly in those patients affected by NAFLD as well [81], thus paving the way for a redox imbalance. The increase in Cu serum levels correlates with viral load [82]. Since Cu overload may lead to several deleterious effects, it is possible to speculate that the Zn therapy is beneficial for HCV patients because it also provokes a reduction in Cu levels, as already described in other pathologies like Wilson's disease [83].

Cu, in fact, is a transition metal extremely harmful because, as well as Fe, it is characterized by an elevated redox potential; thus it may participate in redox reactions, like Fenton's reaction, promoting ROS generation and consequently OS, as already reported in other tissues and pathologies [84]. To avoid its unhindered reactivity, biological systems developed an intricate network of proteins that prevents the existence of free copper. GSH, a key antioxidant, able to suppress Cu toxicity through its binding to this metal, maintains it in a reduced state and avoids its redox cycling [85]. GSH decrease, associated with Cu deregulation, may play a key role in the HCV-related OS onset. The role of Cu in HCV infection and the related OS is probably underestimated. Oxidative potential of Cu on low-density lipoprotein (LDL), in fact, is known for a long time [86]. This event not only has a clinical relevance in cardiovascular risk enhancement, but also may have a deep impact in HCV-related damage, even if some apparent contrasting data are reported. Several studies, in fact, describe the presence of a high level of oxLDLs in HCV infected patients [20, 87], according to the plasma Cu elevation, suggesting a possible role in disease pathogenesis; on the contrary, other papers suggest their potential role in inhibiting the HCV entry in the cells [88, 89]. Nevertheless, why should the virus produce something that could be able to inhibit its entry into cells? Currently, there is not an explanation. It could be a protective response of the organism; the oxidation of LDL, in fact, as a consequence of the inflammatory response activation, could be an attempt to counteract viral infection. Alternatively, it may only be a secondary phenomenon occurring only in a late phase as a result of the prooxidant environment generated by the HCV, also through the imbalance of transition metals homeostasis, such as copper. This latter hypothesis implies that LDL oxidation has actually a low impact in countering the infectivity of HCV, since it occurs only when the virus has already activated its pathological mechanisms. Although the oxidation of LDL is an event well established in HCV patients, the mechanisms underlying it are not well clarified. The topic is intriguing, and the role of Cu needs to be better explained, not only because Cu and oxLDL evaluations may have a deep diagnostic and prognostic impact, but also because a deep comprehension of their involvement in HCV-related disease could open the way to new antiviral approaches.

Altered Cu homeostasis is associated with a reduction of ceruloplasmin [90–92]. This protein is important for both the trafficking of copper, binding 95% of circulating copper [70], and Fe, because, at hepatic level, it is the main ferroxidase enzyme, which is crucial for a proper Fe transport [93]. Thus, its deregulation may also represent a sign of systemic Fe perturbation.

As a matter of fact, patients with chronic HCV infection are commonly characterized by elevated levels of serum ferritin and hepatic iron [8]. Hepatic iron increase could be functional to viral cell cycle; in fact, a recent paper indicates that transferrin receptor 1 (Tfr1) may be important for the cell entry of viral particle [94]. The relevance of this metal is further highlighted by several studies and clinical trials that demonstrate how the hepatic iron levels influence hepatic injury and response to therapy in chronic HCV patients [95–98]. Furthermore, patients with a virus-induced severe grade of hepatitis show higher levels of serum iron with respect to patients characterized by a lower grade of hepatitis. Note that serum iron is positively correlated with intense steatosis, fibrosis, and biochemical and histological parameters, indicating liver inflammation [99].

The mechanism underlying iron deregulation is still unclear; however, in such a context, hepcidin seems to be a key player. Hepcidin is a protein synthesized in the liver where iron, inflammation, and OS promote its expression [100]. This protein, through the modulation of ferroportin, is crucial for the maintenance of systemic iron homeostasis.

Through the use of a mouse model of HCV infection, it was suggested that the hepatic decrease of hepcidin causes an increased intracellular iron storage and, at the same time, OS. This, in turn, may promote the expression of ferroportin in the duodenum and in the macrophages, thus leading to increased iron serum levels [101]. This mechanism could explain the iron derangement in HCV patients. Furthermore, it was recently proposed that the HCV-related downregulation of hepcidin may also exert a deep impact on the virus life cycle, because it seems to possess an antiviral activity [102]. Therefore, through the hepcidin inhibition HCV reaches two goals to save its viral cycle and to promote derangement of iron homeostasis.

The latter event may be crucial in OS-mediated liver injury [103]. In fact, Fe is an extremely reactive transition metal, and an excessive presence may induce mitochondrial injury increasing the risk of HCC development [104]. Accordingly, a Fe reduction therapy has been shown to counteract hepatocyte injury in patients with HCV infection [97], confirming an important role of iron in HCV-related liver injury.

So, the OS induced by HCV promotes the onset of a dangerous loop involving, once again, mitochondrial damage; hence, the deranged homeostasis of metals may enhance ROS production and mitochondrial failure, which may participate in the alteration of metal homeostasis. Mitochondria, in fact, play a key role in the maintenance of Cu and Fe homeostasis [105, 106].

## 5. New Potential HCV Therapeutical Approaches

In the last decade, the gold standard in the HCV treatment was represented by the combination of pegylated interferon (IFN)-*α* and ribavirin. This therapy, administered for 24 or 48 weeks, produced viral suppression in approximately 40–50% of patients infected by HCV genotype 1 and in 80% of those infected by HCV genotypes 2 and 3 [107]. Today, the new therapeutic approach contemplates the use of a triple therapy, (IFN)-*α* and ribavirin plus telaprevir or boceprevir, two direct-acting antiviral (DAA) agents known to be NS3/NS4A protease inhibitors [107]. Unfortunately, although these new treatments reach a sustained viral response (SVR) in 63–75% HCV genotype 1 patients and a reduction in therapy length, persistent limitations to treatment still exist. In particular, many new side effects have been encountered which are in need of adequate management strategies as well as drug interactions, other than the persistence of virus resistance and interferon intolerance [108]. To overcome these problems today new therapeutic treatments are under investigation, such as DAA of second generation, targeting NS5B, or host targeting molecules, like cyclophilin inhibitors, and the use of IFN-free therapy, to reduce intolerance to treatment and to enlarge the potential patients cohorts [107].

The use of molecules counteracting oxidative stress at mitochondrial levels or treatments able to restore a proper metal homeostasis could be really helpful in HCV therapy. Accordingly, in fact, it was recently demonstrated that the use of a cell-permeant iron chelator and GSH ethyl ester decreased oxidative RNA damage, positive selection, and the nucleotide and amino acid substitution rates of HCV [109]; all these events are involved in the virus resistance to the antiviral therapy. Unfortunately, antioxidants and other strategies decreasing ROS/RNS in HCV patients achieved poor effects. In fact, when being used alone, antioxidants like N-acetylcysteine (NAC), vitamin E, or ascorbic acid ameliorate liver damage but did not affect HCV titer [107]; currently, only seldom, the outcome of antiviral therapy was improved when they were used in combination with IFN [107].

The above-mentioned scarce efficacy could be justified by the fact that the canonical antioxidants are not taken up into mitochondria, the major cellular source of damaging free radicals within cells [61]. To decrease specifically mitochondrial oxidative damage, mitochondria-targeted antioxidants have been developed like mitoquinone (MitoQ) [110, 111]. This novel class of compounds combines the antioxidant potential of ubiquinone with a lipophilic triphenylphosphonium cation, which facilitates the mitochondrial storage [111]. Some studies, both *in vitro* and *in vivo*, have shown that the selective mitochondrial accumulation of MitoQ enhances its antioxidant potential if compared to untargeted antioxidants [112]. This molecule, which accumulates in the liver after oral administration [110], was employed in phase II trials against HCV [113]. Despite its scarce impact on the viral load, it was able to decrease the liver damage. It is conceivable to imagine that the great reduction of OS and inflammation, due to its mitochondrial specificity, may be highly detrimental for the virus persistence.

Another class of molecules that gained great interest as antioxidant and mitochondrially targeted antiviral agents is the analogues of cyclosporine A (CsA), for example, the cyclophilin (Cyp) inhibitors [114]. They are nonimmunosuppressive molecules and have a great anti-HCV potential, as demonstrated by *in vivo* and *in vitro* studies [115–117]. The prototype of this class of molecules is Alisporivir (also known as Debio-025 or DEB025) [118]. This drug, besides its ability to counteract viral replication, was demonstrated to prevent the HCV-related mitochondrial respiration dysfunctions, the collapse of mitochondrial membrane potential, the consequent ROS overproduction, and the mitochondrial calcium overload [119]. Cyp inhibitors are host oriented therapeutic, and for this reason, their use in the common clinical practice is difficult because of the risk of cell toxicity; even clinical and experimental data push towards this direction.

Another mechanism through which it is possible to counteract the HCV-related OS is the maintenance or the rescue of a correct metals homeostasis. In fact, the positive effects reached through a Zn supplementation on the HCV-related liver damage were described, although viral titer was not affected [120]. Other reports, indeed, describe that phlebotomy treatment, done to reduce the circulating iron content, produces a 2.95 odds ratio of response to IFN therapy [121]. To date, the potential effect of copper reduction in HCV treatment was not yet described, but we need to keep in mind an interesting issue. Recently, the ability of a wide range of natural polyphenols to counteract various steps of HCV life cycle, like cell entry, replication, or spreading was deeply reviewed [122]. Of particular interest among these molecules are quercetin, (−)-Epigallocatechin-3-gallate (EGCG), and Silymarin/Silibinin [122], because they are known to be powerful antioxidants. However, a property of these compounds, too often underestimated, is their ability to bind reactive metals like Fe and Cu [123, 124]. This behavior is extremely interesting, but to date its relevance in HCV treatment has not been yet investigated. Through the metal binding, in fact, polyphenols could exert their antiviral actions via different mechanisms, because not only they can control metals redox reactivity, counteracting the OS occurrence, but also through the modulation of intracellular metal content, they could hinder the activity of some viral proteins, for example, NS3 or NS5A. It could be imagined that polyphenols, reaching and penetrating the hepatocytes, once bound to Fe or Cu, can cause an intracellular rise of metal levels, which can, in turn, counteract the viral replication. Accordingly, it has been reported that increased intracellular levels of Fe and Cu can create a hostile environment for the life cycle of the virus [125, 126].

On these bases, the use of molecules with an antioxidant potential, targeting mitochondria, or being able to bind metals, could be really helpful to eradicate HCV infection, at least as treatments complementary to gold standard therapies. On the other hand, a deeper knowledge of the mechanisms of action of such compounds can reveal new interesting abilities that could lead to the formulation of new efficient therapies with less side effects and major tolerance.

## 6. Discussion

The hepatitis C virus promotes a prooxidant cellular status through several molecular mechanisms. While at cellular level mitochondria appear to be the most affected organelles, at systemic level the deregulation of trace metals homeostasis is associated with pathological mechanisms involving OS and inflammation, which in turn may be once more correlated with mitochondrial failure. Furthermore, if we consider the fact that HCV infection is associated with a decrease of antioxidant defenses, it leaps to the eyes that the increase of ROS is not efficiently counteracted. Thus, the virus establishes a vicious circle in which the molecules can suffer oxidative damage with the consequent alteration of their physiological functions.

To this regard, an antioxidant therapy could be useful to counteract, at least in part, the pathological consequences caused by HCV-related OS. Unfortunately, until now, antioxidant therapy had scarce effectiveness, either if used alone or combined with interferon (IFN) antiviral treatment [127]. In fact, as previously reported, the main goal reached by antioxidant supplementation is the reduction of OS and inflammatory state caused by HCV, which causes, in turn, a reduction of virus-related liver damage.

On the other hand, we need to keep in mind that HCV induces OS through numerous molecular pathways, for example, mitochondrial damage (Figure 1) and altered metal homeostasis; thus it is difficult to imagine an antioxidant approach with a such wide range of action. Furthermore, it should always be remembered that HCV produces several other effects besides the OS; thus, we think that the antioxidant effects may be helpful to counteract HCV-related damage, but it is likely that they are not so effective if used alone. Probably, the use of antioxidants able to selectively target mitochondria can more efficiently counteract the OS related mitochondrial dysfunctions, thus leading to more powerful healthy effects than those obtained through the use of canonical untargeted antioxidants.

Another potentially effective approach is the use of natural polyphenols to counteract the OS related to HCV infection. Several natural compounds have shown antiviral effects counteracting viral entry (e.g., Honokiol), replication (e.g., Quercetin), or spreading (e.g., Silymarin) [122]. Natural compounds are known to be powerful antioxidants, and some of them are also able to bind Cu and Fe, controlling, in this way, their redox potential [128]. If we consider their abilities, it could be conceivable to imagine that the use of “natural cocktails,” obtained by mixing different compounds, each of which is able to counteract a specific aspect of HCV infection (entry, replication, and OS generation), could be effective.

In conclusion, we believe that despite the poor results obtained so far by using antioxidant therapies, this antiviral therapeutic strategy should not to be set aside.
